# Pulse and pulsating supercharging phenomena in a semi-enclosed pipe

**DOI:** 10.1038/s41598-023-28214-x

**Published:** 2023-01-24

**Authors:** Fei Wang, Heng Li

**Affiliations:** 1 College of Physics, Hexi University, Zhangye, 734000 China; 2 State Key Laboratory of Coal Resources and Safe Mining, China University of Mining and Technology, Xuzhou, 221116 China

**Keywords:** Energy science and technology, Engineering

## Abstract

Considering the discontinuous square pulse wave and continuous sine pulsating wave, we report a distinctive supercharging phenomenon of fluid in a water-filled semi-enclosed pipe and reveal the supercharging regularity. We demonstrate that there can be significant supercharging phenomena at the pipe end-face if the water is periodically injected at the pipe inlet with certain frequency. Compared to the traditional pulsating injection method, the present injection strategy can remarkably enhance the peak pressure of the water at the end face of the pipe. We explained this phenomenon by numerical simulations based on the computational fluid dynamic method. It’s found that there is a quantitative relationship between the optimal pulse frequency, pipe length and wave speed. The proposed frequency model is suitable for the multi-waveform, such as sine wave, square wave and arcuate wave. The fluid pressure at the pipe end-face intermittently increases and the end-face peak pressure is largest when the inlet injection frequency equals to the optimal frequency.

## Introduction

Water wave is a common physical phenomenon in nature and it mainly includes two forms. The first is the external wave in an open space such as the waves in river and ocean^1–3^. The second is the internal wave in a constrained space such as the pressure wave in water-filled pipe^4–6^. The propagation phenomenon of the pressure wave inside a water-filled pipe often appears in engineering such as the water hammer phenomenon^7,8^ and pulse (or pulsating) hydraulic fracturing (PHF) process^9–12^.

Low permeability is an objective property for the most rock formation, which hinders the development of natural gas. To increasing the permeability of rock formation, hydraulic fracturing (HF) method is widely used. However, the fracturing effect is not very good in some situation due to the limited injection pressure. In contrast, the PHF method can improve the hydraulic effects by enhancing the peak pressure during the pulse or pulsating process of pressure wave. Even so, the magnification of peak pressure at end face is usually less than 20% compared to the injected peak pressure at inlet. The main reason may be attribute to the injection way at the pipe inlet, including the injected wave shape, amplitude and frequency, etc. In our recent research^12^, we used the computational fluid dynamic (CFD) method and statistic method find the optimal injection frequency. However, we only focus on the square wave or sine wave in past study. Are there any supercharging phenomena for any injection form? Based on these considerations, we conduct this study and report the distinct finding.

Usually, the pulse wave is discontinuous and pressure jump exists. While, the pulsating wave is continuous and no pressure jump appears. The typical pulse waves include square wave and arcuate wave. The representative pulsating waves include sine wave and cosine wave.

**Figure 1:**
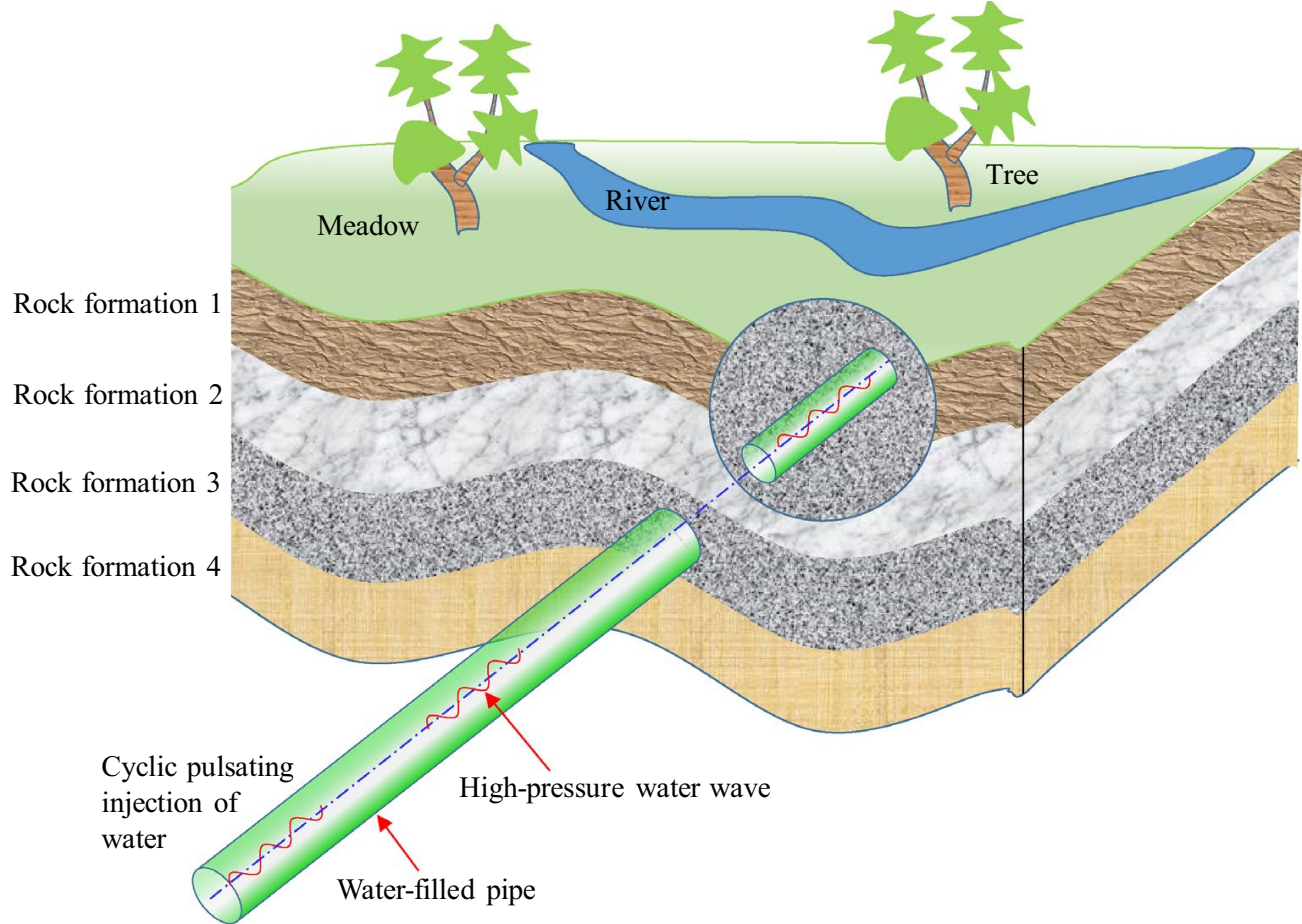
Sketch map of the reservoir stimulation by the pulse hydraulic fracturing method. The injected high-pressure water can fracture the rock during the pulse supercharging process.

Oscillatory injection and discharge of a column of water leads to a pipe water hammer even cavitation^8^. Traditionally, the oscillatory injection and discharge in pipe can increase the local water pressure through the supercharging process, but the supercharging range is very limited. For example, the enhancement of water pressure is less than 20% in the previous PHF studies^9,10^. Objectively, there is huge application potential for further increasing the water pressure. One of the typical application scenarios is the rock formation or reservoir stimulation^10^. As shown in Fig. 1, the pulsating pressure wave is applied at the pipe inlet, the peak pressure of water gradually increases due to the pulsating supercharging process, causing the fracture of the rock.

Pulse or pulsating injection is a potential way to increase the local water pressure by supercharging effect of water hammer. The key is to find the most effective injection way. In other words, it is vital to reveal the influence regularity of pulse parameters on the supercharging phenomenon and process, and then design the best effective injection scheme to achieve the most significant supercharging. Although many previous researches^8–11^ reported the supercharging phenomena, the related water pressure is intermittent constant even attenuated during the supercharging process, causing the limited magnification of pressure. By contrast, we find that there is intermittent increase of pressure if applying certain frequency, giving rise to a remarkable supercharging phenomenon different from the previous findings^12^.

**Figure 2:**
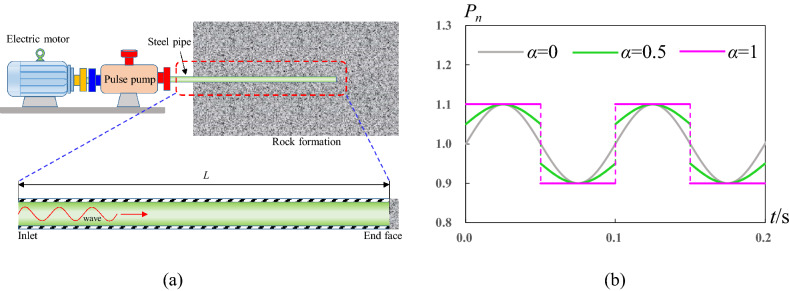
Pulse injection forms and conditions. (**a**) Pulse injection apparatuses and pipe sectional drawing. (**b**) Normalized water pressure applied at pipe inlet. *α* = 0 represents sine pulse injection, *α* = 1 represents square pulse injection, and 0 < *α* < 1 represents arbitrary injection.

To precisely control the pulse injection, the pipe inlet is connected to pulse pump, relief valve, etc. Water is periodically injected and released at left inlet, and the right end face of pipe is a blind end sealed by rock or rigid body as shown in Fig. 2a. Without loss of generality, a straight pipe is considered here and the right end is closed. It can be regarded as one-dimensional flow for the propagation process of pressure wave inside the water-filled pipe during the pulse injection. Therefore, one-dimensional flow model and equation are adopted to research the pulse supercharging phenomena. From the viewpoint of pressure wave as shown in Fig. 2b, the typical injection forms include the sine pulsating injection, square pulse injection, and arcuate pulse injection which is an arbitrary combination of the sine and square wave.

## Method

It’s difficult to compress the water because of its larger elastic modulus. So, in most situations, water is regarded as incompressible media when the water pressure is not very high. For example, in the researches of water wave^13,14^ and liquid drop^15–18^. However, in some situations^19,20^, water should be regarded as compressible media when the water pressure is very high such as in water hammer process where the local water pressure may be far higher than 1 MPa^21^. Objectively, water is weakly compressible media and its compressibility is non-ignorable during pulse supercharging process (PSP). Therefore, the weakly compressible Navier–Stokes (N-S) equations are chosen as the control model to simulate the supercharging phenomenon inside pipe. Considering the one-dimension characteristics of pipe flow in PSP, the weakly compressible N-S equations^22,23^ are adopted as follow.

1 $$\left\{ \begin{gathered} \frac{\partial p}{{\partial t}} + u\frac{\partial p}{{\partial x}} + \rho a^{2} \frac{\partial u}{{\partial x}} = 0 \hfill \\ \frac{\partial u}{{\partial t}} + \frac{1}{\rho }\frac{\partial p}{{\partial x}} + u\frac{\partial u}{{\partial x}} = - \lambda \frac{u\left| u \right|}{{2D}} \hfill \\ \end{gathered} \right.$$

where *p* is the fluid pressure, *u* is the fluid velocity, *ρ* is the fluid density, *a* is the wave speed, *D* is the diameter of pipe, *λ* is the friction drag coefficient. For the laminar flow, the friction drag coefficient is defined by *λ* = 64/*Re*, where *Re* is the flow Reynolds number. For the turbulent flow, the friction drag coefficient is defined by $1/\sqrt \lambda = 2\log ({\text{Re}} \sqrt \lambda ) - 0.8$, which is an implicit expression about *λ*. This implicit formula cannot be directly solved. The Newton iteration method is used to solve the approximate solution of the friction drag coefficient. To concisely show the results, we discuss the water pressure through the nondimensional pressure *P*_*n*_ normalized by the inlet average pressure *P*_*av*_, namely *P*_*n*_ = *p*/*P*_*av*_.

For the simulation of weakly compressible flow, the common methods include the property line method, one-order finite difference, etc. These methods have some disadvantages such as limits of smaller time-step or lower accuracy, and so on. MacCormack method^24^ has two order accuracy in time and space solution, which is a good choice for the simulation of supercharging process, so it is adopted. For all I know, the first-order methods such as the method of characteristics (MOC) can deal with the continuous sine wave, but for the discontinuous square wave, these first-order methods are not applicable due to the poor ability of discontinuity capture. We developed the simulation program based on Fortran90 language, which was validated by experimental data (see Supplemental Material 1, Sec. 1.). The detailed introduction for the validation can be found in Reference^12^, including the simulated region, resolution condition, etc. For the PSP simulation, it is vital to assign the boundary conditions. Because the right end of pipe is blind, the wall boundary is used at the right end face where the velocity and pressure satisfy the reflection condition of wall. For the left pipe inlet, pressure boundary condition is assigned. Continue pulse of sine or square or their arbitrary combination is applied at pipe inlet as shown in Fig. 1b. The normalized pressure function is expressed by unified form as follow

2 $$P_{n} {(}t{) = (1 - }\alpha {)}A \cdot {\mathbf{sin}}\left( {2\pi ft} \right) + \alpha A \cdot {(} - {1)}^{{{\mathbf{mod}}{(2}t{/}T{)}}} + 1$$

where *A* is the pulse amplitude, *f* is the pulse frequency which is related to the pulse period *f* = 1/*T*, **sin**() represents the sine function, **mod()** represents the integer function. Weight coefficient *α* = 0 represents that periodic sine pulse, *P*_*n*_(*t*) = *A*•**sin**(2*πft*) + 1, is applied at inlet, *α* = 1 represents that periodic square pulse, *P*_*n*_(*t*) = *A*•(-1)^**mod**(2*t*/*T*)^ + 1, is applied at inlet, 0 < *α* < 1 represents that arbitrary combination of sine and square is periodically applied at inlet. It is noteworthy that *P*_*n*_ is normalized by the average pressure *P*_*av*_.

## Supercharging phenomena

**Figure 3:**
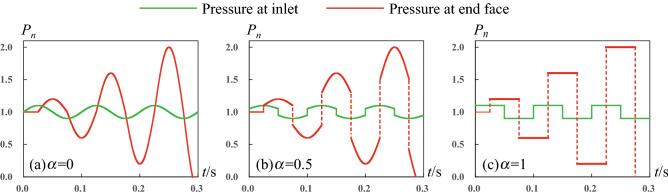
Pulse supercharging phenomena based on the reliable numerical computation. The green line is the injected water pressure at pipe inlet, the red line is the water pressure at pipe end face. Here the pulse frequency is *f* = 10 Hz, pipe length is *L* = 25 m, (**a**) *α* = 0, (**b**) *α* = 0.5, (**c**) *α* = 1.

When water is injected in the form of pulse, there are propagation, reflection and superposition of pressure wave inside pipe. It is found that the peak of water pressure at pipe end face intermittently increases as shown in Fig. 3. These phenomena indicate that there is supercharging process inside the pipe, causing the intermittent magnification of water pressure. Supercharging phenomena are observed for various pulse or pulsating injection forms including sine wave, square wave and so on. In above case, the pipe length is 25 m, pipe diameter is 0.1 m. At the pipe inlet, the average pulse pressure is 1 MPa, the normalized pulse amplitude is 0.1, pulse frequency is 10 Hz, and the wave speed is 1000 m/s. When changing the pipe parameters and pulse parameters, we still observed the supercharging phenomena (see Supplemental Material 1, Sec. 2). We find that the supercharging phenomenon is very significant only at some certain pulse frequencies. Besides, the supercharging phenomenon would be restrained if increasing pipe length or decreasing pipe diameter due to the wall friction drag. The supercharging phenomenon indicates that it is practicable to enhance the local water pressure by controlling the inlet pulse or pulsating wave.

The supercharging phenomenon can be explained from the viewpoint of propagation and reflection of pressure wave in a water-filled pipe. Without loss of generality, we also take the above ca se as an example to explain. The incidence wave travels from the inlet to the blind end and then reflects. At this moment, the water pressure of end face increases due to the energy transformation from fluid kinetic energy to pressure energy. This process is similar to the water hammer process in the pipe. Then, the reflected pressure wave reversely travels to the inlet. Based on the inlet boundary control of flow, some water is released. Then, larger flow rate is applied and the new incidence wave forms which has larger impact energy. The local water pressure further increases when the incidence wave reaches the blind end and reflects again. Afterwards, above process repeats, so the peak pressure intermittently increases. Different from the square pulse wave shown in Fig. 3c and arcuate wave shown in Fig. 3b, the sine pulsating injection (Fig. 3a) provides consecutive pressure variation where numberless incidence waves are applied at pipe inlet. As a result, the supercharging process provided by the sine pulsating is more consecutive compared to the jumping supercharging process given by the square pulse. To further show the supercharging process, two detailed pressure evolution movies are provided in supported material 2 (see Supplemental Material 2, Video 1 and Video 2).

## Supercharging regular pattern

The supercharging phenomenon does exist in water-filled pipe, but it appears only at certain frequency. For example, when frequency is 1 Hz, we didn’t observe supercharging phenomena. When the inlet pulse frequency is set to 20 Hz, the supercharging phenomenon at end face is weak and the normalized maximum peak pressure is only 1.2. We tested thousands of cases with different pipe length, wave speed, pulse amplitude, and pulse frequency. We find that the supercharging phenomenon is remarkable only at certain pulse or pulsating frequencies. For the most of frequencies, the supercharging phenomenon is not apparent.

**Figure 4:**
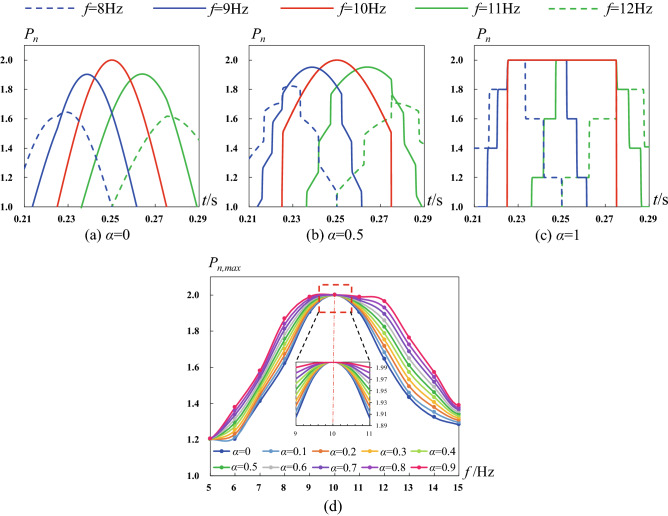
Variation regularity of the normalized water pressure near the third crest at pipe end face. (**a**) Pressure–time curves when *α* = 0, (**b**) Pressure–time curves when *α* = 0.5, (**c**) Pressure–time curves when *α* = 1, (**d**) Peak pressure of the third crest when varying frequency *f* and weight coefficient *α*.

Taking above case as an example, the pipe length is 25, wave speed is 1000 m/s. We monitor the third pressure crest at pipe end face when applying different pulse frequencies. As shown in Fig. 4a,b, we find that the peak pressure provided by *f* = 10 Hz is largest, indicating that the supercharging phenomenon is most significant at *f* = 10 Hz when *α* < 1. More detailed results are shown in Fig. 4d which shows that the maximum pressure appears at *f* = 10 Hz. For the square pulse case (*α* = 1), the largest peak pressures are same for different frequencies (8–12 Hz), but their duration times are different. As shown in Fig. 4c, the frequency *f* = 10 Hz produces the longest duration time of peak pressure. Based on above analysis, we can see that frequency *f* = 10 Hz provides the best supercharging effect.

**Figure 5:**
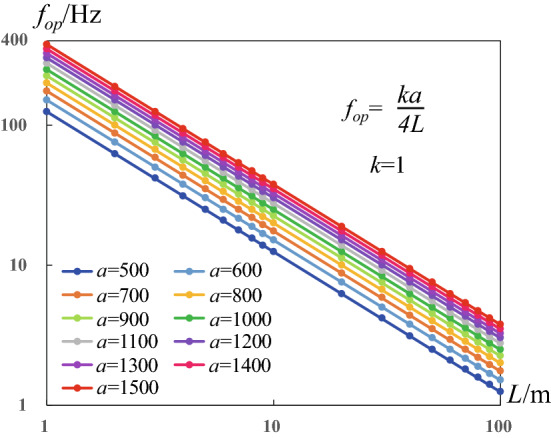
Distribution law of optimal pulse frequency. The optimal pulse frequency satisfies *f*_*op*_ = *ka*/(4*L*), where taking *k* = 1 as an example to show.

Based on thousands of cases, we tested the influence of pipe length, diameter, wave speed, pulse amplitude, frequency, wave shape on the supercharging phenomenon and process (see Supplemental Material 1, Sec. 2). We find that the supercharging effect at pipe end face has positive correlation with the pulse amplitude, and has negative correlation with the pipe length due to the wall friction drag. Besides, decreasing the pipe diameter will restrain the supercharging effect. We find that there is a family of frequencies at which the supercharging effect is best. Namely, the inlet impulsive excitation provides the largest peak pressure or longest duration time of peak pressure at these optimal pulse frequencies, which provide the most significant supercharging effect. The optimal pulse frequencies are shown in Fig. 5.

It is found that there is a quantitative relationship between the optimal pulse frequency, pipe length and wave speed. The quantitative relationship is *f*_*op*_ = *ka*/(4*L*) where *k* is arbitrary positive odd. This universal formula shows that the optimal pulse frequency *f*_*op*_ is proportional to wave speed *a* and inversely proportional to pipe length *L*. More detailed verification is given in Supplemental Material 1, Sec. 2. To determine the coefficient *k*, we computed and recorded the third peak pressure at pipe end-face when changing *k* from 0 to 10, with a very small increase. Then, the *P*_n,max_-*k* function can be obtained by the local quadratic polynomial fitting. The location of local peak of P_n,max_-k function represents the *k* value, which provides the best supercharging effects.

## Discussion on the supercharging mechanism

The supercharging phenomena have been shown in above sections. However, the supercharging mechanism has not been revealed and discussed in detail. To clarify the inner mechanism of supercharging, we discuss the supercharging process and analyze the supercharging principle by studying the transient evolution characteristics of the pressure and velocity.

It is not necessary to discuss every case with different pipe length, wave speed, and so on. In contrast, it is meaningful that focusing a certain case to research the supercharging process from the time and space dimensions. Without loss of generality, we choose a sine-pulsating case where the pipe length is 250 m, wave speed is 1000 m/s, the amplitude of sine-pulsating is 0.1*P*_*n*_. Besides, the drag effect of wall is not considered temporarily, which does not influence the optimal frequency if *a* = constant confirmed in above section.

To recreate the supercharging process, according to the formula *f*_*op*_ = *ka* /(4*L*_*x*_), the pulsating frequency is set to 1 Hz for the present case where *L*_*x*_ = 250 m and *a* = 1000 m/s. The pulsating period is *T* = 1/*f* = 1 s. The other conditions are same as those mentioned in above section, including the initial and boundary conditions.

During the computation, the initial fluid pressure is set to 1 MPa in whole pipe, the average pressure of sine-pulsating is also 1 MPa and the amplitude of pulse is 0.1 MPa at inlet. In following discussion, we used the normalized pressure *P*_*n*_ to describe the pressure characteristics of fluid, where *P*_*n*_ = 1 represents the real fluid pressure *P* = 1 MPa.

**Figure 6:**
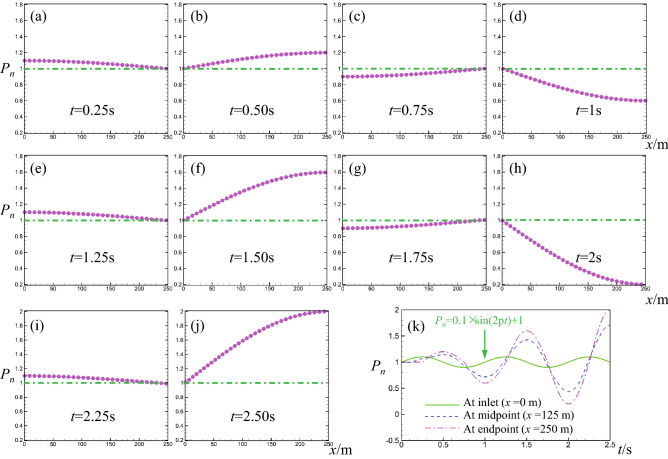
Normalized pressure-position curves alone pipe axes direction during 2.5 pulsating periods where the pipe length is 250 m and the pulsating frequency is 1 Hz^25^.

At *t* = 0 s, the normalized pressure is 1 in whole pipe. The detailed evolution of the pressure is shown in Fig. 6a–j. At *t* = 0.25 s, the pressure increases to 1.1 at inlet (*x* = 0 m). Here, 0.25 s is 1/4 pulsating period. According to the wave speed *a* = 1000 m/s, the pressure wave just right travels to the end face (*x* = 250 m) at *t* = 0.25 s. At this moment, a 1/4 sine wave forms as shown in Fig. 6a. Then, during the second 1/4 period (0.25–0.5 s), the inlet pressure gradually decreases to 1, and the end face pressure gradually increase to 1.2 shown in Fig. 6b. It is easy to understand the decrease of the inlet pressure because it is controlled by the inlet boundary condition. While the pressure *P*_*n*_ = 1.2 at the end face indicates that there is a supercharging process, causing the magnification of pressure at end face. At *t* = 1.5 s, the magnification is more apparent and the pressure reaches 1.6 at the end face (Fig. 6f). At *t* = 2.5 s, the pressure further increases to 2 at the end face (Fig. 6j). The detailed temporal evolution of the pressure is given in Fig. 6k.

From Fig. 6k, we seen that the inlet pressure (at *x* = 0 m) periodically change in a way of sine and its peak pressure is constant, which equals to 1.1. However, the peak pressure at the end face (*x* = 250 m) periodically increases because of the periodic magnification of amplitude at the end face. The peak pressure increases from 1.2 at *t* = 0.5 s to 1.6 at *t* = 1.5 s, and then further increases to 2 at *t* = 2.5 s. Similar phenomena are observed at the midpoint (*x* = 125 m), but the magnification effect is weaker than the one at the end face.

**Figure 7:**
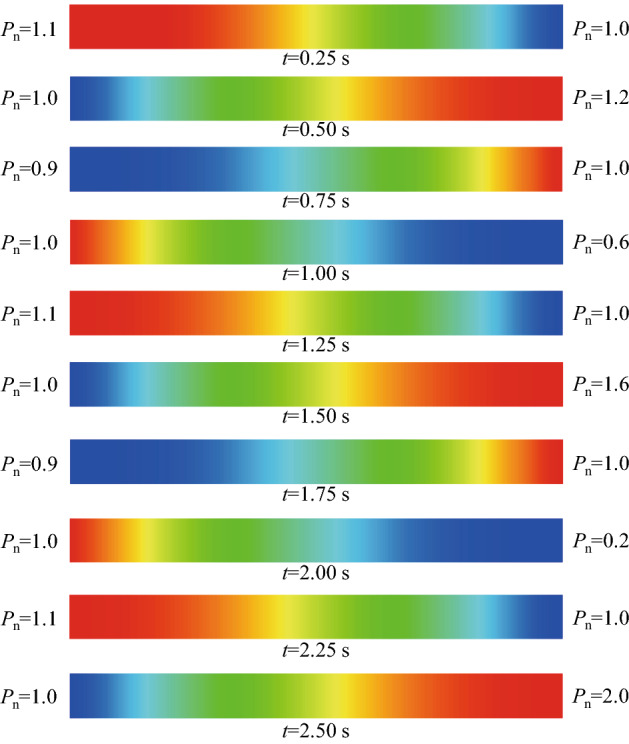
Contours of the normalized water pressure in the water-filled pipe during 2.5 pulsating periods where the pipe length is 250 m and the pulsating frequency is 1 Hz.

The more intuitive pressure distribution and evolution are shown in Fig. 7. We can see that the water pressure periodically pulses at the pipe inlet varying between 0.9 and 1.1. Similarly, the water pressure also periodically pulses at the pipe end face, but the peak pressure of water intermittent increases at the pipe end face varying from 1 to 2. The injected pressure wave continuously changes at the inlet, so the pressure distribution is continuous along the pipe axes direction.

For the sine-pulsating case, the injected pressure wave is reflected at the pipe end face due to the impermeability of the blind end. In fact, the supercharging process is continuous because of the continuity of injected pressure waves and their reflection. For the square pulse case, the supercharging process is not continuous due to the discontinuity of injected pressure waves and their reflection. Although the continuities of the supercharging process are different, the supercharging mechanisms are same. The key reason of the supercharging phenomenon is that the increasing injected dynamic energy is periodically supplied at the pipe inlet, which converts into the pressure energy of the water at the blind end face of the pipe. The supercharging effect is best when the injection is at the optimal pulse frequency, at which the wave offset is not obvious and the conversion efficiency of kinetic energy is highest. Therefore, it is suggested that adopting the optimal pulsating frequency to achieve the supercharging aim.

## Conclusion

The internal wave propagation is a ubiquitous phenomenon in nature and engineering. In this report, we report the pulse and pulsating supercharging phenomenon induced by the internal pressure wave in semi-closed pipe. We find that a remarkable supercharging phenomenon exists at the pipe end face. We also find that there is a family of optimal frequencies, *f*_*op*_ = *ka*/(4*L*), at which the supercharging effect is very significant. The optimal frequency was obtained by the statistical analysis of peak pressure on the basis of many CFD simulations. However, we are surprised to find that the optimal injection frequency of water is equal to the natural vibration frequency of water column in a pipe. At these optimal frequencies, the peak pressure of fluid at pipe end-face intermittently increases. These supercharging phenomena can be observed for any injection waveform, including the pulse injection and pulsating injection. This work reveals the pulse and pulsating supercharging phenomena and process in a water-filled pipe.

## Supplementary Information

Supplementary Information 1.

Supplementary Video 1.

Supplementary Video 2.

## Data Availability

The data that support the findings of this study are available from the corresponding author upon reasonable request.
