# Supplementary material for: Pulse and pulsating supercharging phenomena in a semi-enclosed pipe
Source: Sci Rep. 2023 Jan 24;13:1332. doi: 10.1038/s41598-023-28214-x (PMC9873632; doi:10.1038/s41598-023-28214-x)
Supplement: Supplementary file 1 — Supplementary Information 1. [file 41598_2023_28214_MOESM1_ESM.pdf]

Supplemental Material 1

Section 1: Simulation program validation by theory and experiment

Objectively, there is strong similarity between the pulse supercharging process (PSP) and water hammer, where the water can be regarded as the weakly compressible medium. Therefore, the present algorithm and program are examined by the water-hammer theory in an ideal situation ignoring the friction drag, then they are further validated by two classical water-hammer experiments considering the friction drag.

During the water-hammer process, there is a theoretical solution if ignoring the wall friction drag and assuming that both ends of pipe are blind after the closure of valve. As an example, the initial static water head is 30m and the flow velocity is 0.1m/s inside a smooth pipe. Then, the right valve closes at t=0s and the water-hammer appears. The wave speed is assumed as a=1000m/s. Based on these conditions, we conduct the simulation and monitor the pressure characteristics at the pipe end face. The results (Fig. 1) show that the water head at end face periodically pulses and its pulsation amplitude is 10.2m. The theoretical increase of water head can be assessed by $a|\triangle u|/h$=10.2m. The present numerical solution agrees well with the theoretical solution as shown in Fig. 1.

We also validated the present algorithm and program by comparison with the experimental data. The first experiment focused on the laminar flow at Reynolds number $\boldsymbol{Re}$=1870 inside a pipe with length 37.23m and internal diameter 2.21cm. The initial static head at inlet is 32m, which keeps constant during the experimental process. The initial flow velocity is u_0=0.1m/s and the valve closure time is at t=0.009s. The wave speed is a=1319m/s when the water temperature is 15.4°C.

Results are shown in Fig. 2(a) and Fig. 2(b). It is seen that the water head (or pressure) periodically fluctuates at the middle point and endpoint due to the reflection of pressure waves. The present computation results agree well with the experimental results (Bergant et al., 2001). Particularly, the present peak and frequency of water head obtained from the numerical computation are consistent well with the experimental data, indicating that the present algorithm and program can give accurate simulation results.

The second experiment was also conducted in above pipe with the same size including length and diameter. But the initial flow velocity is larger than the first case. The flow velocity is 0.3m/s, and the Reynolds number is 5600. For this case, the flow is turbulent instead of laminar. The simulation is conducted based on these parameters. Results are shown in Fig. 2(c) and Fig. 2(d). It is found that the present numerical results agree well with the experimental data (Bergant et al., 2001). These comparisons further confirm that the present calculation program is accurate and credible.

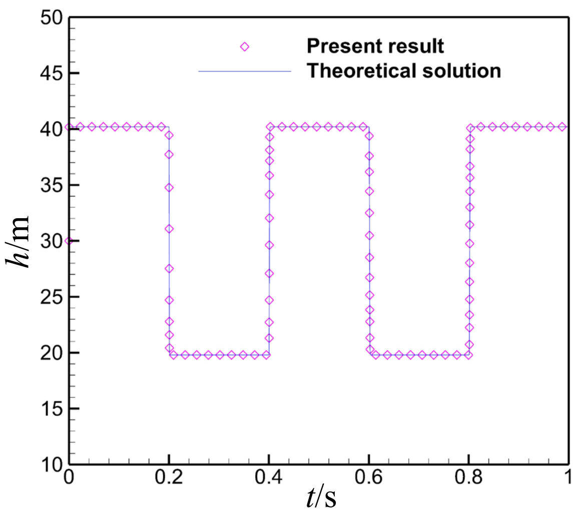

FIG. 1. Comparison of water heads between the present numerical simulation and theoretical solution at the valve (endpoint) when the initial velocity is 0.1m/s. The water pressure p is related to water head h, and there is a relationship $p=\rho gh$.

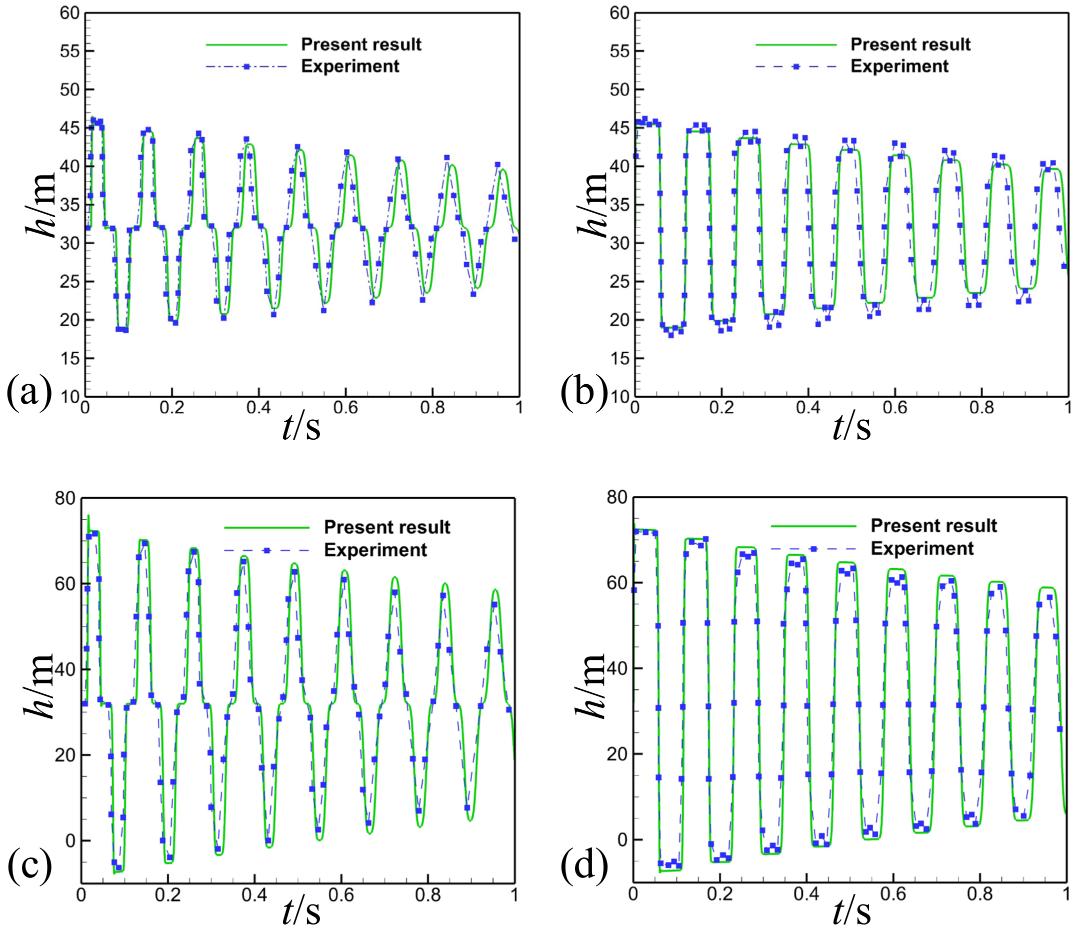

FIG. 2. Comparison of water heads between the present simulation and experimental data, (a) evolution of water head at the midpoint when u_0=0.1m/s, (b) evolution of water head at the valve (endpoint) when u_0=0.1m/s, (c) evolution of water head at the midpoint when u_0=0.3m/s, (d) evolution of water head at the valve (endpoint) when u_0=0.3m/s. (Reference: A. Bergant, A.R. Simpson, and J. Vtkovsk, Developments in unsteady pipe flow friction modelling, J. Hydraul. Res. **39**(3), 249 (2001)).

Section 2: Influence of pipe diameter, length, wave speed and pulse amplitude

In pipe flow, there is friction drag on the wall. The wall friction drag is positively correlated with Reynolds number. From the definition of Reynolds number $\boldsymbol{Re}=\rho uD/\mu$, we can see that the Reynolds number is proportional to fluid density, velocity, pipe diameter, and inversely proportional to fluid viscosity. For the present PSP, the fluid velocity is very small in most regions of pipe because the net flow rate is zero, which is caused by the blind end of pipe. But, the local fluid velocity is significant because of travelling pressure wave, causing a larger local Reynolds number. Therefore, the wall friction drag affects the supercharging process to a certain degree.

Without loss of generality, we choose three pipe diameters, including D=0.005m, 0.01m, 0.1m. The other conditions are same, including pipe length (L=25m), pulse shape (sine), pulse frequency (f=10Hz), wave speed (a=1000m/s), etc. As shown in Fig. 3, results show that the supercharging phenomena exist in above three kinds of pipes. However, the supercharging phenomenon is restrained by the pipe diameter or wall friction drag. When decreasing the pipe diameter, the maximum peak pressure decreases and the supercharging effect decreases. The reason is that the decease of pipe diameter enhances the local friction drag, which restrains the supercharging process. Even though, the supercharging phenomena still exist when applying the optimal pulse frequency.

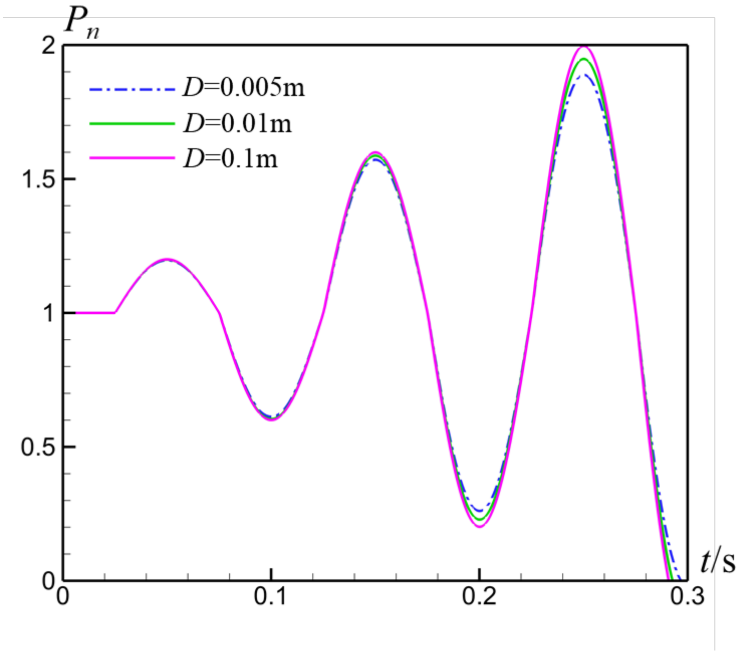

FIG. 3. Normalized pressure-time P_n-t curves at pipe end face when considering the influence of wall friction drag by adjusting pipe diameter, where L=25m and f=10Hz.

We conducted thousands of cases and examined the supercharging phenomena in various conditions. We find that the supercharging phenomena exist in long and short pipes, as shown in Fig. 4. When the pipe length is 5m, the supercharging phenomenon is significant at frequency f=50Hz. However, when the pipe length is 25m, the supercharging phenomenon is significant at frequency f=10Hz. At f=100Hz, the supercharging phenomenon is not significant. At f=1Hz, there is no supercharging phenomenon.

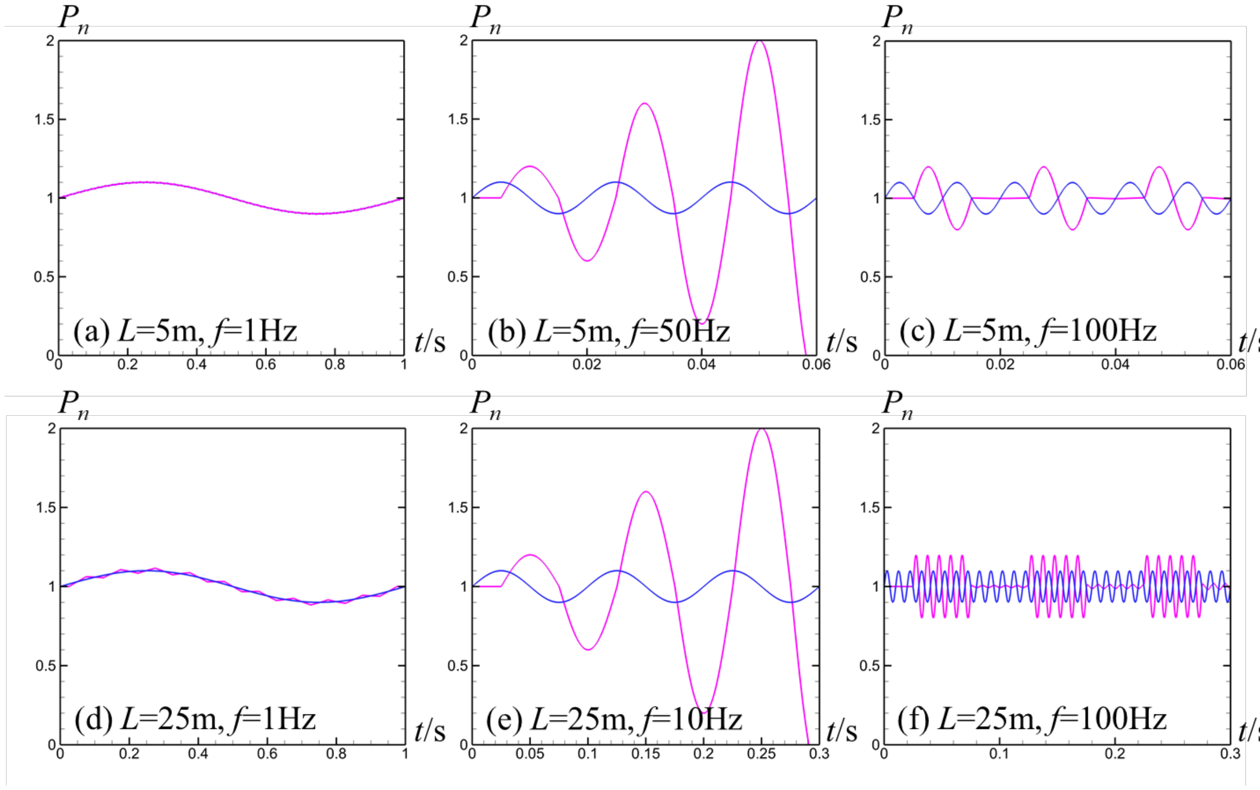

FIG. 4. Normalized pressure-time P_n-t curves at pipe inlet and end face, (a) L=5m, f=1Hz, (b) L=5m, f=50Hz, (c) L=5m, f=100Hz, (d) L=25m, f=1Hz, (e) L=25m, f=10Hz, (f) L=25m, f=100Hz. Here, the blue line represents the pressure-time curve at pipe inlet, the pink line represents the pressure-time curve at pipe end face.

The travelling speed of pressure wave is related with the physical properties of fluid, material of pipe and rock, also related with the geometric features of pipe. The definition of wave speed is $a = c/\sqrt{(1+4E_w/(DgK)}$, where c is the sound velocity in water and it equals to 1476m/s at 20°C, E_w is the elastic modulus of water and it equals to 2.18×10^3MPa, K is the resistance coefficient. In fact, the resistance coefficient K is a complex function of pipe elastic modulus, pipe diameter, pipe wall thickness, rock elastic modulus, Poisson ratio, etc. Hence, it is senseless and tedious to analyze every influence factor when discussing the influence of wave speed on the supercharging phenomena. Instead, it is practicable to give a range of wave speed and then discuss the effects of wave speed in this range. Because the wave speed always falls within a range no matter how to adjust and combine the related influence factors. In view of these reasons, we discuss the effect of wave speed in a practical range

from a=500m/s to a=1500m/s.

As an example, the pipe length is set to 25m. For every wave speed, the normalized pressure-time curve is obtained from low-frequency to high-frequency. Partial results are shown in Fig. 5. We find that the supercharging phenomenon at pipe endpoint (end face) is significant at f=8Hz when the wave speed is 800m/s. The most remarkable supercharging phenomena also appear at f=10Hz and f=12Hz when the wave speed is 1000m/s and 1200m/s, respectively.

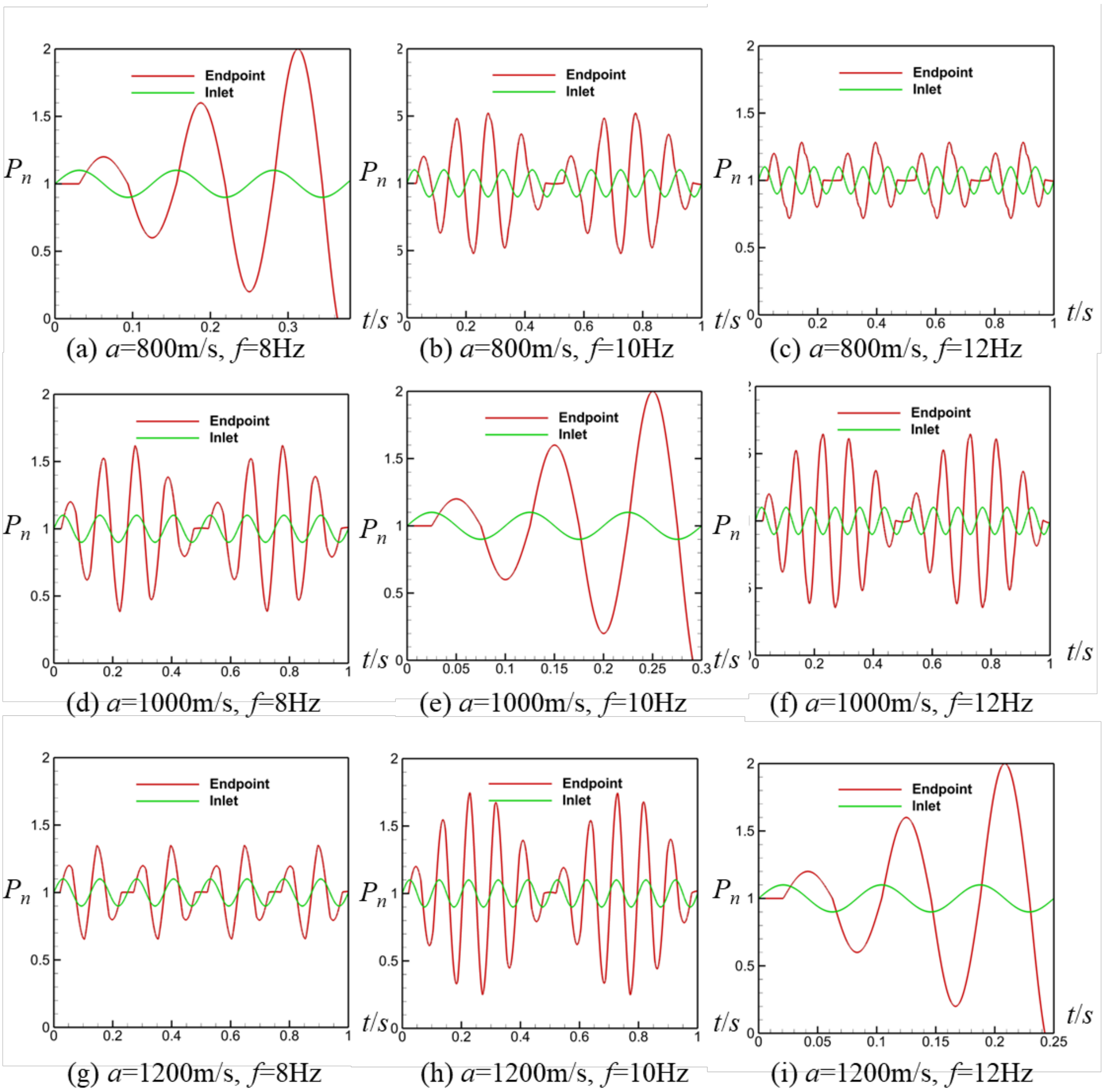

FIG. 5. Normalized pressure-time curves where the pipe length is L=25m. The upper part (a, b, c) is obtained when the wave speed is 800m/s, the middle part (d, e, f) is obtained when the wave speed is 1000m/s, the lower part (g, h, i) is obtained when the wave speed is 1200m/s.

The pulse amplitude is another important parameter affecting the supercharging phenomena. Our results show that the magnification of peak water pressure is positively correlated with the pulse amplitude as shown in Fig. 6. Namely, we can enhance the supercharging effect by increasing the pulse amplitude at pipe inlet.

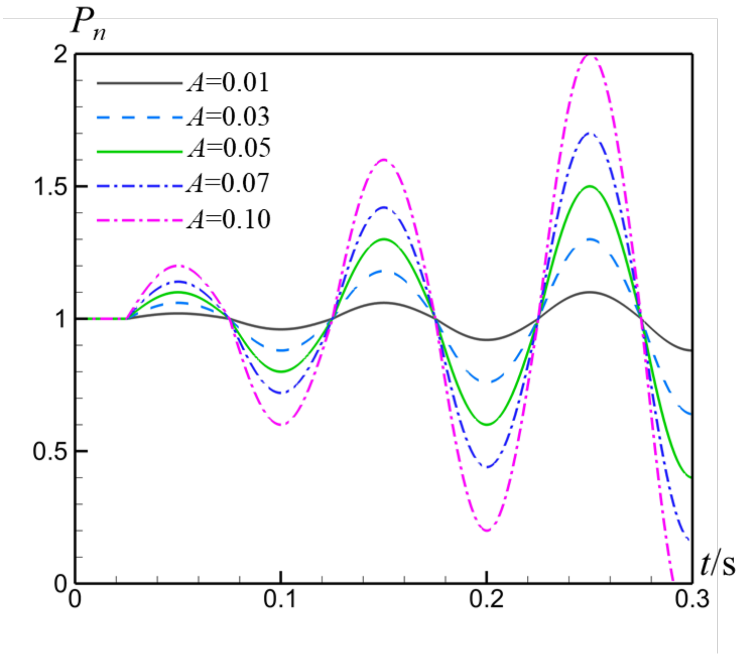

FIG. 6. Normalized pressure-time curves at pipe end face when considering the influence of pulse amplitude, where L_x=25m, f=10Hz, and a=1000m/s.
